# Impact of racism and discrimination on physical and mental health among Aboriginal and Torres Strait islander peoples living in Australia: a systematic scoping review

**DOI:** 10.1186/s12889-021-11363-x

**Published:** 2021-07-03

**Authors:** Camila A. Kairuz, Lisa M. Casanelia, Keziah Bennett-Brook, Julieann Coombes, Uday Narayan Yadav

**Affiliations:** 1 Department of Public Health, Torrens University, Sydney, Australia; 2 The George Institute for Global Health, Sydney, Australia; 3 Centre for Primary Health Care and Equity, Faculty of Medicine and Health, UNSW, Sydney, Australia; 4 Center for Research Policy and Implementation, Biratnagar, Nepal

**Keywords:** Aboriginal and Torres Strait Islanders, Mental health, Physical health, Racial discrimination, Racism, Scoping review, Wellbeing

## Abstract

**Background:**

Racism is increasingly recognised as a significant health determinant that contributes to health inequalities. In Australia efforts have been made to bridge the recognised health gap between Aboriginal and Torres Strait Islander people and other Australians. This systematic scoping review aimed to assess, synthesise, and analyse the evidence in Australia about the impacts of racism on the mental and physical health of Aboriginal and Torrens Strait Islander peoples.

**Methods:**

A systematic search was conducted to locate Australian studies in English published between 2000 and 2020. Five electronic databases were used: PubMed, CINAHL, Embase, Web of Science and the Australia’s National Institute for Aboriginal and Torres Strait Islander Health Research. The search strategy included a combination of key words related with racism, mental health, physical health and Indigenous people. Data were extracted based on review questions and findings were synthesized in a narrative summary.

**Results:**

Of total 338 searched studies from five databases, 12 studies met the inclusion criteria for narrative synthesis where eight were cross-sectional studies and four prospective cohorts. General mental health and general health perception were the most frequently studied outcomes followed by child behaviour, smoking and substance consumption and specific health conditions. The prevalence of racism varied between 6.9 and 97%. The most common health outcomes associated with racism were general poor mental health and poor general health perception. More specific health outcomes such as anxiety, depression, child behaviour, asthma, increased BMI and smoking were also associated with racism but were analysed by a limited number of studies. Three studies analysed psychological distress, negative mental health, sleeping difficulties and negative perceived mental health according to severity of exposition to racism.

**Conclusion:**

Racism is associated with negative overall mental and negative general health outcomes among Aboriginal and Torres Strait Islander peoples. Strategies to prevent all forms and sources of racism are necessary to move forward to bridging the health gap between Aboriginal and Torres Strait Islander peoples and non-Indigenous Australians. Further research is needed to understand in more detail the impact of racism from an Aboriginal and Torres Strait Islander definition of health and wellbeing.

**Supplementary Information:**

The online version contains supplementary material available at 10.1186/s12889-021-11363-x.

## Background

Racism has been defined as the manifestation of racist beliefs, emotions, behaviours, and practices that perpetuate and aggravate disparity of opportunity of an ethnoracial group [1]. Racism has been described to occur at three main levels [2]. These levels include a) interpersonal racism, which refers to the discriminatory behaviour from one individual towards another, b) systemic or institutional racism, expressed by institutions through policies or practices that reduce benefits and opportunities among the oppressed group [3], and c) internalized racism defined as the adoption by the stigmatized people of negative beliefs about their capacity and value [4]. Globally, research is demonstrating with increasingly solid evidence that racism and discrimination have detrimental impacts on health and is a significant factor contributing to health disparities in different countries [5, 6]. Although the exact pathophysiological process involved in racism affecting health is not completely understood, it has been demonstrated that racism causes alteration in some physiological activities that lead to permanent changes associated with disease [7]. It can also reduce access to other important determinants of health such as employment, housing, and education [8] and can lead to unhealthy behaviours such as smoking and alcohol consumption [9]. Global efforts against any kind of discrimination have been made and are reflected through conventions and declarations such as the international convention on the elimination of all forms of discrimination [10], the Universal Declaration of Human Rights [11] and, the United Nations Declaration on the Rights of Indigenous Peoples [12]. Despite this, racism towards indigenous peoples continues to be consistently reported worldwide [13–16].

Australia is inhabited by two different groups of Indigenous peoples: Aboriginal and Torres Strait Islanders. Each has its own distinctive and established values, cultural protocols, and unique living manner [17]. The term “Indigenous” is often used to describe both Aboriginal and Torres Strait Islander peoples. However, many Aboriginal and Torres Strait Islander people dislike being referred to as “Indigenous” as the term homogenises two different cultural groups into one word that has also been used to describe flora and fauna [18]. Within this manuscript, we capitalise the term Indigenous according to terminology preferences when referring to Aboriginal and/or Torres Strait Islander peoples set by the Council of Social Services [19]. The word “Indigenous” refers to Indigenous Australians whilst the word “indigenous” is used to describe all indigenous peoples of the world. Discrimination and racism against Aboriginal and Torres Strait Islander people in Australia is a current reality aggravated by shared experiences of dispossession and intergenerational trauma from ongoing colonial practices [20, 21]. The 2014–2015 National Aboriginal and Torres Strait Islander Social Survey, evidenced that one-third of Aboriginal and Torres Strait Islander people aged 15 or older had felt treated unfairly in the previous year because of being Aboriginal and/or Torres Strait Islander [22]. According to the Reconciliation Barometer survey, 43% of Aboriginal and Torres Strait Islander respondents experienced racial prejudice in the last 6 months compared to 20% of the rest of the community [23]. A recent study available in the Journal of Australian Indigenous Issues revealed that three in four people unconsciously hold a negative prejudice against Aboriginal and Torres Strait Islander peoples [24].

In Australia, the “Indigenous health” panorama is well known for the existence of what is called by the government and public health sector as “The gap.” It refers to the wide health disadvantage that Aboriginal and Torres Strait Islander peoples have in comparison to the rest of Australians. The Australian Institute of Health and Welfare estimated that Aboriginal and Torres Strait Islander people born between 2015and 2017 had 8.6 and 7.8 fewer years of life expectancy than other male and female Australians, respectively [25]. In the year 2011, Aboriginal and Torres Strait Islander people experienced 2.3 times the total disease burden (disability-adjusted life years or DALYs) of other Australians [26], they also have nearly three times the risk of being physiologically distressed and two times the risk to die by suicide than other Australians [27]. A statement of intent called the “Close the Gap initiative” was signed in 2008 by the prime minister of the time to narrow the health disparity experienced by Aboriginal and Torres Strait Islander peoples by the year 2030 [28]. However, 10 years after the initiative started, follow-up showed negligible progress, and the program is very far from achieving its goals [29]. In 2020, the Closing the Gap targets were refreshed after many years of advocacy from Aboriginal and Torres Strait Islander communities and organisations to widen the focus. The Closing the Gap refresh now includes four “priority reform areas” and 16 new targets; however, it has been met with a critique on the ability for communities to enact true self-determination and lacks focus on structural reform needed to address racial disparities [30, 31].

Nationally and internationally, academics have argued that failure of efforts to bridge health inequalities has been due to interventions focusing primarily on behaviour change at individual and interpersonal levels despite the solid evidence recognising the relationship of structural determinants of health such as racism and health disparities [32–34]. The “2030 Agenda for Sustainable Development” by the United Nations and the Australian Medical Association have recognized the need to address indigenous health inequalities and have made a call to increase indigenous peoples participation and reduce institutional and interpersonal racism [35, 36]. Studies have already showed that initiatives addressing racism have indeed the potential to improve health [37]. However, solid evidence of the unique characteristics of Aboriginal and Torres Strait Islander peoples is needed to move forward to create and implement more effective interventions in Australia [38]. Consequently, this scoping review aims to localize and analyse evidence in regard to the magnitude of racism and its association with multiple physical and mental health outcomes among Aboriginal and Torres Strait Islander peoples. It aims to provide a precise description of racial discrimination and its impact on the physical and mental health of Aboriginal and Torres Strait Islander peoples. The results will serve as foundation for researchers, policymakers and, change-makers to design and implement culturally tailored public health interventions to tackle this issue and hence, improve the persistent health inequalities between Aboriginal and Torres Strait Islander peoples and other Australians.

## Methods

The protocol for this scoping review was previously completed and published [39]. The reporting guidelines and criteria set in Preferred Reporting Items for systematic review (PRISMA) [40] were also followed and a PRISMA extension for Scoping Reviews (PRISMA-SCR) checklist was filled (Additional file 1**).**

This review followed the stages proposed on the methodological framework for scoping reviews by Arksey and O’Malley [41] and later revised by Levac [42]. The reviewe involved six phases explained below:-.

### Stage 1. Identifying the research question

The aim of this study is to understand and analyse the impacts of racism on the health of Aboriginal and Torres Strait Islander people living in Australia.

### Stage 2. Identifying relevant studies

A systematic search was conducted between between the 01st of January of 2000 and June 2020 using the following databases: the Australia’s National Institute for Aboriginal and Torres Strait Islander Health Research, CINAHL, PubMed, Embase and Web of Science. This timeframe was decided based on findings evidencing that no primary studies are lost when performing searches that include studies from the last 20 years [43]. We Used a combination of different keywords related to “Aboriginal and Torres Strait Islander peoples”, “Racism”, “Discrimination” and “health”, using “OR” and “AND”. The complete list of key words is shown in Table 1. Additionally, citation tracking was performed using the reference lists of all selected studies and a manual search was conducted using Google and institutions websites relevant to the topic.

**Table 1:** List of search terms

**#1.** Racism OR Discrimination OR “Racial Prejudice(s)” OR “Racial discrimination” OR “Covert racism(s)” OR Harass OR Bully OR “Unfair treat” OR Oppress. **AND** **#2.** “mental health” OR Depression OR Anxiety OR stress OR Distress OR Suicide OR “quality of life” OR “self-efficacy” OR “satisfaction with illness” OR “satisfaction with life” OR “Psychological distress” OR “emotional problems” OR “Psychological illness.” **OR** **#3.** “physical health” OR “wellbeing” OR “cancer” OR “cardiovascular disease” OR “blood pressure” OR “Hypertension” OR “dysfunctional breathing” OR “Respiratory difficulties” OR “Chronic Obstructive Pulmonary Disease” OR “disease “OR “Life satisfaction” OR “Quality of life” OR “BMI” OR “Body max index” OR “Asthma” OR “Cardiovascular disease” OR “Diabetes” OR “Blood pressure” OR “Hypertension” OR “Heart disease” OR “chronic conditions” OR “Chronic disease” OR smoking OR tobacco OR “Alcohol” OR Drug OR “Substance use.” **AND** **#4.** Indigenous OR “Indigenous people(s)” OR Aboriginal OR “Torres Strait Islander” OR “First people(S)” **AND** **#5.** Australia OR “Rural Australia” OR “Remote Australia” OR “Urban Australia”	

### Stage 3: study selection

All the search results were exported to the citation manager EndNote X9 [44] and duplicates were removed. Then, tittles and abstracts were screened independently by two reviewers to select relevant papers. Full- text articles meting inclusion criteria were assessed independently by two reviewers. Discrepancies between reviewers was resolved by discussion to reach consensus.

#### Inclusion criteria

i. Studies in English conducted among Aboriginal and Torres Strait Islander peoples of all ages and gender regardless of any socio-demographic characteristics.
  Perceived experience of racism defined as the feeling of receiving an unequal valuation or unfair treatment for being an Aboriginal or Torres Strait Islander person. The experience of racism could be self-reported or reported by a child’s carer or a witness such as family or friends. Papers analysing health outcomes with racism exposure at any point in time before the study were included.
ii. Outcomes measuring physical and mental health spheres along with other health-related outcomes. Physical health outcomes included chronic conditions such as, cardiovascular disease, chronic obstructive pulmonary disease (COPD) and asthma, hypertension, diabtes, cancer and abnormal body mass index. It also included health risk behaviours (smoking, alcohol consumption and other substance use). Mental health outcomes including psychological factors like depression and anxiety, psychological distress, social and emotional wellbeing, illness representations (satisfaction with life, quality of life, self-efficacy, satisfaction with illness) and suicide.
iii. Only studies using quantitative methods (control-case studies, cross-sectional, and cohort studies) were included.

#### Exclusion criteria

i. Articles studying autoimmune disorders, and cystic fibrosis. (This decision was based on i) The multifactorial cause of such conditions and their strong polygenetic component [45–47] which we consider is an important limitation to draw conclusions about a causal association and ii) The lack of studies assessing the association between racism and autoimmune disorders found during our pilot search in the Australian context.)
ii. Experimental studies (randomized controlled trials (RCTs) and controlled clinical trials (CCTs)).
iii. Studies published in language different to English, studies found only as abstract, letters to the editor, commentaries, editorials and reviews.

### Stage 4. Charting the data

Primary investigator extracted the data into an excel spreadsheet. The following information was extracted when available: author, year of publication, journal, type of study (study design), location of the study (state or city), sample size, sample source, sample demographic characteristics, exposure measure including tools or instruments, exposure timeframe and severity, studied outcomes, strength and direction of the associations between racism and health outcomes along with the type of data used to quantify the association (odds ratio, hazard ratio, correlation coefficients). UNY randomly selected 10% of the articles meeting eligibility criteria to double check all the data listed before. Data was compared, and any discrepancies were resolved by consensus.

### Stage 5. Collating, summarizing and reporting the results

The data of the studies included was analysed and summarized following a descriptive synthesis process based on the recommendations of the Joanna Briggs Institute Reviewer’s Manual [48]. Quality appraisal of the studies included in the review was performed using the Joanna Briggs Institute critical appraisal tools, according to each study design [49, 50].

### Stage 6. Consulting with key stakeholders

Two members of this review team (authors KBB and JC) who represent Aboriginal and Torres Strait Islander people living in Australia reviewed and validated the interpretations of the data.

## Results

**Fig. 1:**
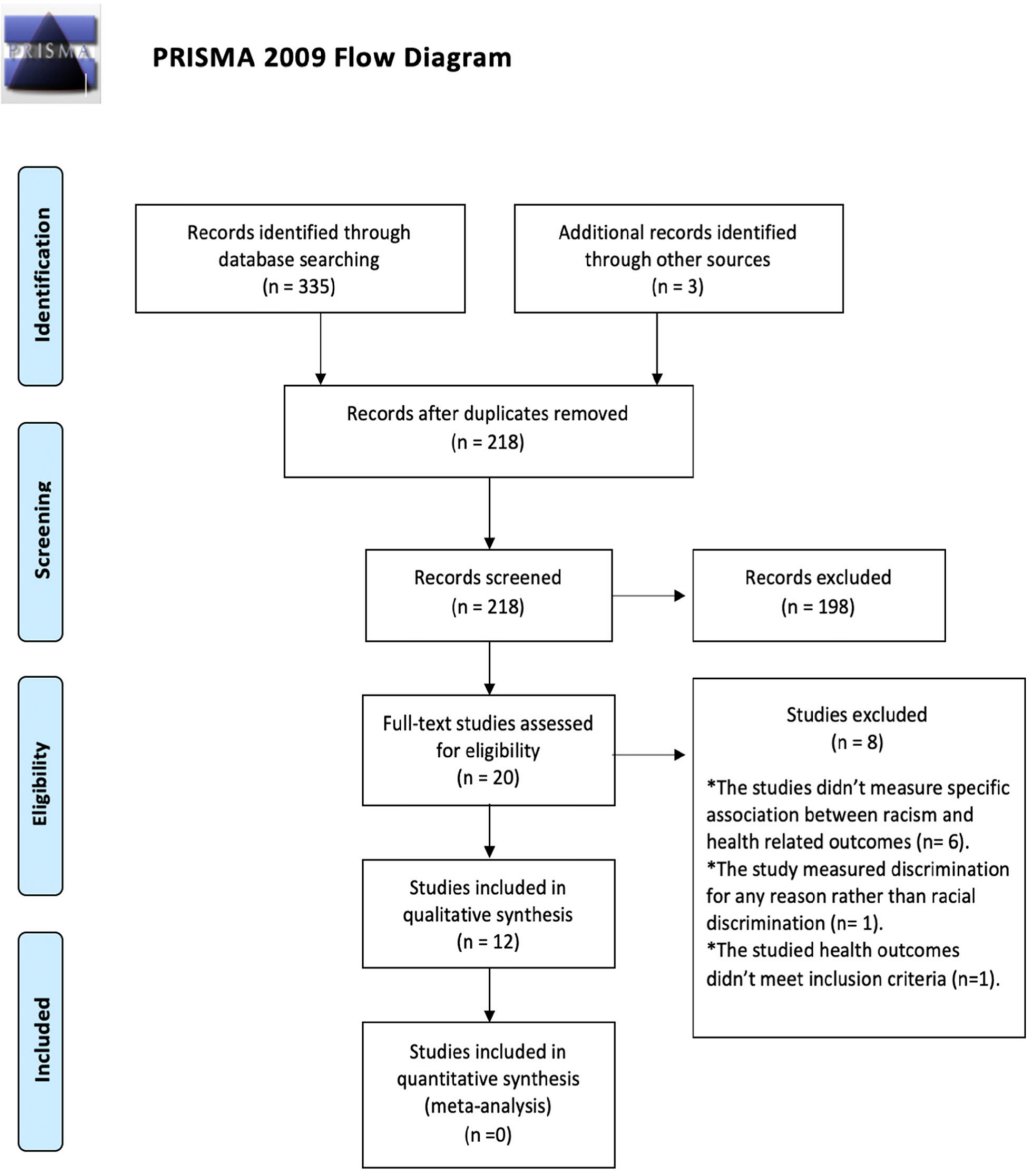
PRISMA 2009 Flow Diagram

After searching on the five databases, 335 articles were found and three more were located by manual search. From these, 120 duplicates were removed, and 218 papers were initially screened. Of 218 titles and abstracts, 20 studies were eligible for inclusion in this review. After full-text assessment 8 more articles didn’t meet inclusion criteria and were excluded as indicated in the PRISMA flow diagram **(**Fig. 1).

### Study characteristics

Of 12 studies included, five (5/12, 41.%) were conducted across different locations within Australia [51–55], Three (3/12, 25%) were conducted in the Northern Territory [56–58], three (3/12, 25%) in Victoria [34, 59, 60], and one (8.%) in an isolated rural town with a non-specified location [3]. Fifty percent (6/12) of the studies included were published after the year 2015 [34, 51–55], another 42 % (5/12) between 2010 and 2015 [56–60], and 8 % (1/12) were published before 2010 [3]. Most studies (8/12, 66.%) were Cross-sectional studies [3, 34, 53, 56–60], whilst four (4/12, 33%) were prospective cohorts [51, 52, 54, 55]. Seven studies were conducted among teens over 12 years and adults [3, 34, 56–60]; four focused on children younger than 12 years [51, 52, 54, 55], and one on elderly people [53]. The total sample size across the 12 articles consisted of 44,517. The article with the smallest sample included 164 participants [57], whilst the larger sample included 2606 participants [53]. It is important to mention that more than one article used the same study for sampling in some cases. Four articles extracted their sample from the Longitudinal Study of Indigenous Children (LSIC) [51, 52, 54, 55], and two articles used the Diabetes and Related conditions in Urban Indigenous people in the Darwin region (DRUID) study [56, 57]. The sample source of each article and the characteristics of the studies are described in Table 2.

**Table 2:** Characteristics of the studies

Study ID	Tittle	Journal	Study Design	Location	Sample size	Sample source	Age group
Cave et al. (2019) [47]	Caregiver-perceived racial discrimination is associated with diverse mental health outcomes in Aboriginal and Torres Strait Islander children aged 7–12 years.	International Journal for Equity in Health	Cohort study	Australia/ 11 cities	1759/ No representative	First 8 waves of The Longitudinal Study of Indigenous Children (LSIC) study	4–12 years
Paradies et al. (2012) [52]	The DRIUD study: exploring mediating pathways between racism and depressive symptoms among Indigenous Australians.	Social psychiatry and psychiatric epidemiology	Cross- sectional study	Darwin (Northern Territory)	185/No representative	Diabetes and Related conditions in Urban Indigenous people in the Darwin region (DRIUD) study	15 years and over
Paradies et al. (2012) [53]	The DRIUD study: racism and self-assessed health status in an indigenous population	BMC public health	Cross- sectional study	Darwin (Northern Territory)	164/No representative	Diabetes and Related conditions in Urban Indigenous people in the Darwin region (DRIUD) study	15 years and over
Macedo et al. (2019) [48]	Effects of racism on the socio-emotional wellbeing of Aboriginal Australian children	International Journal for Equity in Health	Cohort study	Australia/ 11 cities	1060/ No representative	Children of waves 6,7 and 8 of the LSIC study	6–12 years
Temple et al. (2019) [49]	Experiences of Racism among Older Aboriginal and Torres Strait Islander People: Prevalence, Sources, and Association with Mental Health	Canadian Journal on Aging	Cross- sectional study	Remote and non-remote areas across all Australia	2606/Representative	National Aboriginal and Torres Strait Islander Social Survey (NATSISS)	> 45 years
Kelaher et al. (2014) [55]	Experiencing racism in health care: the mental health impacts for Victorian Aboriginal communities	Medical Journal of Australia	Cross- sectional study	2 rural and 2 metropolitan areas of Victoria	755/No representative	Mental health impacts of racial discrimination in Victorian Aboriginal communities’ survey	> 18 years
Shepherd et al. (2017) [50]	The impact of racial discrimination on the health of Australian Indigenous children aged 5–10 years: analysis of national longitudinal data	International Journal for Equity in Health	Cohort study	11 sites across Australia	1239/No representative	Waves 1–6 of the LSIC study	5–10 years
Larson et al. (2007) [57]	It’s enough to make you sick: The impact of racism on the health of aboriginal Australians	Australian and New Zealand Journal of Public Health	Cross- sectional study	one Isolated rural town in Australia	639/No representative	Survey conducted in late 2003	> 18 years
Markwick et al. (2019) [34]	Perceived racism may partially explain the gap in health between Aboriginal and non-Aboriginal Victorians: A cross-sectional population-based study	Ssm-Population Health	Cross- sectional study	People living in private dwellings across Victoria	33,833 people, including 387 Aboriginal Participants	Three Victorian Population Health Surveys (VPHS) conducted in 2011,2012, 2014	> 18 years
Cave et al. (2019) [51]	Racial discrimination and the health and wellbeing of Aboriginal and Torres Strait Islander children: Does the timing of first exposure matter?	Ssm-Population Health	Cohort study	Australia/ 11 cities	1759/No representative	first 8 cohorts of Footprints in time: The Longitudinal Study of Indigenous Children (LSIC)	6 months - 12 years
Priest et al. (2011) [56]	Racism and health among urban Aboriginal young people	BMC public health	Cross- sectional study	Melbourne	172/No representative	Wave 1 of the Young People’s Project (YPP)	12–26 years
Priest et al. (2011) [59]	Racism as a determinant of social and emotional wellbeing for aboriginal Australian youth	Medical Journal of Australia	Cross- sectional study	Top end of Northern Territory	345/No representative	Wave 3 of the Aboriginal Birth Cohort (ABC) study	16–20 years

### Exposure to racism

All articles studied the effect of direct racism except one study that also studied the indirect effect of racism on the child where racism was experienced by the parent or carer [54]. Eight of the included papers studied interpersonal racism [3, 51, 52, 54–57, 59], one study included both interpersonal and institutional racism [53] and three had broad measures of racial discrimination, without considering specific types of racism [34, 58, 60]. The majority of the included papers (10/12, 83.%) studied exposure to racism through questions about having experienced bullying, discrimination, or unfair treatment because of being Aboriginal or Torres Strait Islander [3, 34, 51–55, 58–60];whilst two [56, 57] used the Measure of Indigenous Racism Experiences (MIRE) [61]. In most of the articles, the instrument used (8/12, 66.%) didn’t specify the exposure- timeframe [51, 52, 54–58, 60], whilst a 12- months exposure timeframe was used by three (25%) [34, 53, 59] and only one [3] used a 4-week timeframe. The instruments used to study the exposure to racism along with the time and severity of the exposition are summarized in Table 3. The prevalence of at least one self-reported experience of racism or racism reported by the parents or carer was variable with one study reporting a prevalence of 97% [59]. The prevalence of racism reported by each study and across the different States and Territories and age groups is shown in Table 4 and Table 5. According to the studies included, the state with the highest prevalence of racism was Victoria with reports of prevalence ranging from 52 to 97%. Moreover, first exposure to racism was reported to occur as early in life as 4–5 years of age [55] and according to the studies, people between 12 and 45 reported racism most frequently.

**Table 3:** Instrument to measure racism, type, severity and time of exposition

Study ID	Tittle	Type of racism and direct or indirect	Instrument to measure racism	Time of exposition	Severity of Exposition
Cave et al. (2019) [47]	Caregiver-perceived racial discrimination is associated with diverse mental health outcomes in Aboriginal and Torres Strait Islander children aged 7–12 years.	Interpersonal/direct, caregiver perceived	Has the kid been bullied or treated unfairly at preschool or school by children or adults because they were Aboriginal?	No specified	No specified
Paradies et al. (2012) [52]	The DRIUD study: exploring mediating pathways between racism and depressive symptoms among Indigenous Australians.	Interpersonal/ direct, self-reported	Measure of Indigenous Racism Experiences (MIRE)	No specified	Number of Settings
Paradies et al. (2012) [53]	The DRIUD study: racism and self-assessed health status in an indigenous population	Interpersonal/ direct, self-reported	Measure of Indigenous Racism Experiences (MIRE)	No specified	Number of Settings
Macedo et al. (2019) [48]	Effects of racism on the socio-emotional wellbeing of Aboriginal Australian children	Interpersonal/ direct, caregiver perceived	Has the child been bullied or treated unfairly at school because he/she is Aboriginal or Torres Strait Islander?	-No information on timing of racism exposure. -Cohort K outcomes measured 2 years after info of racism collected. -Cohort B outcomes measured 1 year after info of racism was collected	No examined
Temple et al. (2019) [49]	Experiences of Racism among Older Aboriginal and Torres Strait Islander People: Prevalence, Sources, and Association with Mental Health	Interpersonal and Institutional / direct, Self-reported	In the last 12 months have you had any of the following experiences because you are Indigenous? (prompts card with options displayed) In which situation where you treated unfairly? How often?	In the previous 12 months	Frequency: always, often, sometimes, rarely, once
Kelaher et al. (2014) [55]	Experiencing racism in health care: the mental health impacts for Victorian Aboriginal communities	Interpersonal / direct, self-reported	Survey about types of interpersonal racism experienced in the past 12 months and in what settings	In the previous 12 months	No specified
Shepherd et al. (2017) [50]	The impact of racial discrimination on the health of Australian Indigenous children aged 5–10 years: analysis of national longitudinal data	interpersonal/ indirect and direct, reported by carer	Have you been treated unfairly, discriminated or treated badly because you are Aboriginal or TSI? How often does your family experience racism, discrimination or prejudice? has the study child been bullied or treated unfairly at school because he/she is Aboriginal or TSI?	No specified	Time limited: just in one wave Persistent: in multiple waves
Larson et al. (2007) [57]	It’s enough to make you sick: The impact of racism on the health of aboriginal Australians	Interpersonal / direct, self-reported	Within the past four weeks have you felt emotionally upset as a result of how you were treated because of your race? Within the past four weeks have you experienced any physical stress or symptoms as a result of how you were treated because of your race?	Within the past 4 weeks	No specified
Markwick et al. (2019) [34]	Perceived racism may partially explain the gap in health between Aboriginal and non-Aboriginal Victorians: A cross-sectional population-based study	No specified / direct, self-reported	How often, if at all, have you received unfair treatment in the last 12 months because you are an Aboriginal or Torres Strait Islander? In the last 12 months, have you experienced discrimination or been treated unfairly because of your racial, ethnic, cultural or religious background?	In the previous 12 months	Never, at least yearly, at least monthly: association with health not reported only for Aboriginals
Cave et al. (2019) [51]	Racial discrimination and the health and wellbeing of Aboriginal and Torres Strait Islander children: Does the timing of first exposure matter?	Interpersonal/ caregiver perceived	Has the kid been bullied or treated unfairly at preschool or school by children or adults because he/she is aboriginal? has the kid been bullied or treated unfairly? If yes was it for being Aboriginal or Torres Strait Islander?	No specified	No examined
Priest et al. (2011) [56]	Racism and health among urban Aboriginal young people	No specified / direct, self-reported.	Do you feel discriminated against because you are Koori? (Not at all and a little / some, quite a bit or a lot)	No specified	Amount: analysis with outcomes not reported
Priest et al. (2011) [59]	Racism as a determinant of social and emotional wellbeing for aboriginal Australian youth	No specified / direct, self-reported.	Have you been treated unfairly or discriminated against because you are Aboriginal? (little bit/fair bit and lots)	No specified	Amount: analysis with outcomes not reported

**Table 4:** Prevalence of self-reported racism or racism reported by parent or carer according to Australia States and Territories and age group

	Prevalence range %	Number of studies (%)
**Australian State or Territory**		
Across Australia	6.9–45%	6 (50%)
Northern Territory	32–75%	3 (25%)
Queensland	No studies Found	No studies Found
New South Wales	No studies Found	No studies Found
Victoria	52.3–97%	3 (25%)
South Australia	No studies found	No studies found
Western Australia	No studies found	
Tasmania	No studies found	No studies found
**Age Group**		
Children (0–12 years old)	6.9–20.4%	4 (33.33%)
Teens and Adults (12–45 years old)	32–97%	7 (58.33%)
Elderly (> 45 years old)	31%	1 (8.33%)

The table shows prevalence of self-report of at least 1 experience of racism.

**Table 5:** Prevalence reported racism according to each study

Study	Title	Reported prevalence
Cave et al. (2019) [51]	Caregiver-perceived racial discrimination is associated with diverse mental health outcomes in Aboriginal and Torres Strait Islander children aged 7–12 years.	20.4% at least one exposure
Paradies et al. (2012) [56]	The DRIUD study: exploring mediating pathways between racism and depressive symptoms among Indigenous Australians.	• Frequency: 50% hardly ever, 25% sometimes, often or very often. • Setting: 25% only one setting,30% two settings, 25% three settings, 20% four or more settings.
Paradies et al. (2012) [57]	The DRIUD study: racism and self-assessed health status in an indigenous population.	• Frequency: 50% hardly ever, 25% sometimes, often or very often. • Setting: 25% only one setting, 30% two settings, 25% three settings, 20% four or more settings.
Macedo et al. (2019) [52]	Effects of racism on the socio-emotional wellbeing of Aboriginal Australian children.	• Cohort K 15% • Cohort B 14%
Temple et al. (2019) [53]	Experiences of Racism among Older Aboriginal and Torres Strait Islander People: Prevalence, Sources, and Association with Mental Health.	• 31% At least one experience of racism. • Frequency: 5.7% always, 15.7% often, 39.9% sometimes, 28.1% rarely, 10.6% only once.
Kelaher et al. (2014) [59]	Experiencing racism in health care: the mental health impacts for Victorian Aboriginal communities.	• Frequency: 97% at least one incident, 25% between 1 and 7 experiences, 38% between 8 and 11, 34% 12 or more experiences. • Setting: 67% shops, ^**a**^59% public spaces ^**a**^29.3% health settings.
Shepherd et al. (2017) [54]	The impact of racial discrimination on the health of Australian Indigenous children aged 5–10 years: analysis of national longitudinal data	• Experienced by carers: 40% (69% time limited, 31% persistent). • Experienced by families: 45% (60% time limited, 40% persistent) • Experienced by child:14% (72% time limited, 28% persistent)
Larson et al. (2007) [3]	It’s enough to make you sick: The impact of racism on the health of aboriginal Australians.	Aboriginal people 3.6 times more likely to report racially based negative treatment than non-Aboriginal people **(**^a^**)**
Markwick et al. (2019) [34]	Perceived racism may partially explain the gap in health between Aboriginal and non-Aboriginal Victorians: A cross-sectional population-based study.	Prevalence of racism among Indigenous people not reported
Cave et al. (2019) [55]	Racial discrimination and the health and wellbeing of Aboriginal and Torres Strait Islander children: Does the timing of first exposure matter?	• First exposure to racial discrimination at 4–5 years 6.9% • First exposure at 7 years 8.3% **(**^b^**)**
Priest et al. (2011) [60]	Racism and health among urban Aboriginal young people	Racism was reported by 52.3%
Priest et al. (2011) [58]	Racism as a determinant of social and emotional wellbeing for aboriginal Australian youth	Racism was reported by 32%

**(**^**a**^**) Unstandardised linear regression:** self-reported negative racially based treatment for Aboriginal respondents −3.6. confidence interval 95% (−6.4 - -0.7). The article does not report % prevalence

**(**^**b**^**)** The values 6.9 and 8.3% represent the proportion of participating children who experienced a first exposure to racial discrimination at 4–5 years or at 7 years respectively. The article does not report the general prevalence of racial discrimination among the sample

### Studied outcomes

Fifty percent (6/12) of the articles studied mental and physical health outcomes [3, 54, 55, 57, 58, 60], 33 % studied mental and behavioural outcomes [52, 53, 56, 59], whilst one article examined self-reported general health [34] and another studied mental, physical, behavioural outcomes and smoking or alcohol consumption together [51].

#### Mental health and behavioural outcomes

Among the mental health component, negative general mental health was the most reported outcome (10/12, 83.%) [3, 51–55, 57–60] followed by depression (3/12, 25%) [56, 58, 60], sleeping difficulties (3/12, 25%) [51, 54, 55], bad behaviours (measured in two studies [51, 55] by carer’s report of having been contacted by the school because the child had bad behaviour and in one [52] using the items of conduct problems, hyperactivity and peer problems from the strengths and difficulties questionnaire) (3/12, 25%) [51, 52, 55], and anxiety (2/12, 16.%) [52, 58]. Smoking and alcohol consumption was examined by one study [51] as well as suicide [58].

#### Physical health outcomes

Regarding the physical health component, its association with experiences of racism was mainly studied through the assessment of general health perception (7/12, 58%) [3, 34, 51, 54, 55, 57, 60]. Four articles (33.%) studied the association with Body Mass Index (BMI) [51, 54, 55, 58], one examined Waist-to-hip ratio [58] and one asthma [54].

### Significant associations

#### Mental health and behavioural outcomes

Racism was associated with a negative overall mental health component in 100% of the studies that reported the outcome (10/10) [3, 51–55, 57–60]. Two studies of three repoted association of depression with racism [56, 58] (66%). Studies that assessed bad behaviours [51, 52, 55] and sleeping difficulties [51, 54, 55] showed its association with racism (3/3, 100%), whilst only half of the studies showed association of anxiety with racism(1/2, 50%) [58]. One study that examined behaviours such as alcohol and cigarette consumption among children aged 4–12 years, found association of racism with having tried cigarettes but not with alcohol consumption [51].

#### Physical health outcomes

Almost 43% of the papers studying general health perception found that a negative health perception is associated with racism (3/7, 42%) [3, 34, 60]. One out of four studies [51] examining BMI found that racism is associated with obesity (1/4, 25%). The study that analysed specific conditions found association between asthma and reported racism [54].

#### Association between level of exposure to racism and outcomes

Three articles studied the association between racism and health outcomes according to the severity of the exposure [34, 53, 54]. Temple et al., (2020) found the participants who reported experiencing racism always had a higher risk of presenting psychological distress compared to the ones reporting racism sometimes [53]. A study by Shepherd et al., (2017) found that children were more likely to present negative mental health outcomes and sleep difficulties when the carer reported persistent racism exposure compared to one-time exposure [54]. The results of the study conducted by Markwick et al., (2019) showed that the likelihood of reporting negative perceived health was three times higher when experiencing racism monthly and 1.5 more likely when experiencing racism yearly [34].

### Quality assessment

The quality of the 12 studies included was assessed using The Joanna Briggs Institute critical appraisal tools for cohort and cross-sectional studies [49, 50]. This tool has been considered a valid approach to assessing the methodological quality of studies in systematic reviews [62, 63]. Two reviewers appraised the articles independently (authors CK and UNY) and discrepancies were resolved by discussion to reach consensus. Six studies were classified as high quality, whilst the other six met criteria for moderate quality. The results of the assessment are found in Table 6.

**Table 6:**
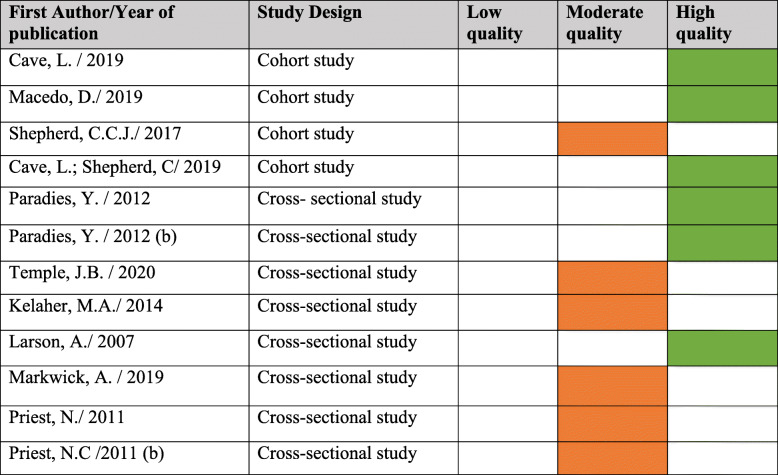
Quality appraisal of the studies using the Joanna Briggs institute Critical Appraisal tools according to study design

**Cohort Studies:**

**∙ Low quality:** studies meeting up to 3 items.

**∙ Moderate quality:** studies meeting 4–7 items.

**∙ High quality:** Studies meeting 8–11 items.

**Cross-sectional studies:**

**∙ Low quality:** studies meeting up to 3 items

**∙ Moderate quality:** studies meeting 4–6 items.

**∙ High quality:** studies meeting 7–8 items.

## Discussion

Aboriginal and Torres Strait Islander peoples have survived and thrived over millennia and are the world’s longest continuing living civilization. Since colonisation, however, disempowerment and ongoing manifestations of racism have undermined Aboriginal and Torres Strait Islander peoples [8, 22–24, 32, 58, 64, 65]. Addressing racism as a determinant of ill health has been identified as a national priority [28, 58] and a paramount component to effectively closing the health gap between Aboriginal and Torres Strait Islander and other Australians that previous efforts have failed to do during the last 10 years [32–36]. Accordingly, this study intended to collect and synthesize available evidence on the impacts of racism on Aboriginal and Torres Strait Islander people’s mental and physical health, which adds to the evidence base for First Nations communities within Australia. Our findings may guide the design and implementation of strategies to reduce racism and therefore contribute to improving Aboriginal and Torres Strait Islander people’s health. With our results, efforts can be directed towards better understanding health racial inequities in particular mental health and cardiovascular conditions, which have previously demonstrated clear racial disparities in prevalence and progession [66, 67]. If racial discrimination is perceived by the larger society as a health risk factor similar to smoking, obesity or substance abuse, this may promote greater interest in reducing behaviours that unintentionally disadvantage Aboriginal and Torres Strait Islander people.

For Aboriginal and Torres Strait Islander people, health is holistic and understood from a whole of life view that goes beyond the physical to encompass social, emotional, and cultural wellbeing of not just an individual but also the whole community [68, 69]. Differentiated from mental health, the term “social and emotional wellbeing” is more culturally appropriate and recognises that connection to culture, land, spirituality, family, and community. These are important to Aboriginal and Torres Strait Islander peoples and can impact one’s wellbeing [70]. It includes social justice, equity, rights, traditional healing, traditional knowledge, and connection to a country [69].

Current scoping review results are in line with international evidence demonstrating the association of racism with poorer physical and mental health outcomes. In this review, the studies reported the association across different age groups indicating that racism produces detrimental health effects on physical and mental health outcomes of Aboriginal and Torres Strait Islander people at any stage of life. Racism had greater negative effects on the mental health component, especially with negative general mental health which was the most studied outcome [3, 51–55, 57–60]. Association with depression, behaviour problems in children and sleeping difficulties were also consistently reported although only three articles included in this review studied those outcomes which is a limitation. However, multiple studies performed in different countries including the biggest systematic review on the topic, supports the association linking racism with depression, post-traumatic stress and suicidal ideation on ethnic minorities [8, 71–73]. Although further studies could provide a better picture of the association of racism with those and unmask other conditions, our study suggest that tackling racism is necessary to achieve better mental health outcomes among Aboriginal and Torres Strait Islander peoples.

Three out of seven articles (42.%) that studied general health perception found a statistically significant negative association with racism [3, 34, 60]. Only one study found an association with obesity [51], and asthma [54] in children, whilst none of the included articles measured conditions such as blood pressure, diabetes, COPD or cardiovascular disease. The association of racism with these conditions should be interpreted with caution since the limited or inexistent number of articles studying these outcomes may lead to an underestimation. Although the findings show that racism can lead to the detrimental health impact. The scarcity of articles studying more specific health outcomes and an Indigenous paradigm of health and wellbeing indicates that further research is needed to demonstrate that racism is inextricably linked with adverse health outcomes and health inequities among Aboriginal and Torres Strait Islander peoples. A starting point for this is the study by Priest et al., (2020), which found that Australian children from different ethnic minorities, including 18 Aboriginal and/or Torres Strait Islander children, who experienced racism on two or more occasions had increased BMI z-scores, systolic blood pressure, waist circumference and saliva inflammatory markers [74]. Supporting our findings, international studies have demonstrated the association between racism and hypertension among ethnic minorities [75, 76]. More research is needed with strong methodological designs grounded in Indigenous methodologies and taking a human rights-based approach that demonstrates the association of racism with different chronic health conditions and its negative impacts on Aboriginal and Torres Strait Islander peoples’ health across the life course. This would contribute to placing racism on the public health agenda and provide an alternative to the current approach to policymaking that often reinforces racism resulting in health inequities [77].

In the present review, one study reported the association of racism with smoking and alcohol consumption [51]. Studies conducted with Aboriginal and Torres Strait Islander peers in New Zealand, indicate that experiences of racism have been linked with smoking behaviour from early adolescence, hazardous alcohol consumption, binge drinking, and substance abuse [78–81]. A study among African-American youth found that substance abuse was a racism coping strategy [82], however, within an Australian context, this association with racism would need to be considered as an ongoing manifestation of colonisation [83, 84]. These results suggest that reducing racism could positively impact health by reducing hazardous substance use among Aboriginal and Torres Strait Islander peoples.

There is an urgent need to address institutional racism as a strategy in Australia in order to improve the health and wellbeing of Aboriginal and Torres Strait Islanders [85, 86]. Whilst in the present review, most of the studies focused on the health impacts of interpersonal racism; Temple et al. (2020) observed that over 50% of older Aboriginal and Torres Strait Islander people who experienced unfair treatment in health care facilities (52%), service settings (50%) and education (55%) were psychologically distressed [53]. Moreover, although most of the papers studied experiences of direct racism, one study [54] found significant negative health outcomes on children whose carers had experienced racism. Similar findings have been found in other countries [87–89]. This adds evidence on the different mechanisms by which racism can cause poor health and highlights the need for strategies to address all different forms and sources of racism if the health gap wants to be bridge.

The prevalence of racism varied from study to study and was as high as 97%. Aboriginal and Torres Strait Islander people aged 12–45 years represent the group with the highest racism experience. A high prevalence of racism against indigenous people has also been reported in other developed countries [90, 91]. Looking at the situation from the other side of the coin, helps to explain the data. In Australia, a study including non-Indigenous Australians aged 25–44 evidenced that 1 in 5 people admit they would discriminate against Aboriginal and Torres Strait Islander peoples in some situations, 30–40% have negative stereotypes of Aboriginal and Torres Strait Islander peoples and 24–30% understand acts of discrimination against Aboriginal and Torres Strait Islander peoples as an automatic response [92]. This illustrates that effective interventions to reduce the health gap must not be directed towards changing Aboriginal and Torres Strait Islander peoples’ behaviors but needs to include a change in beliefs and behaviours of all Australians.

There were five studies conducted across all of Australia [3, 51–55]; however, the study settings were not clearly mentioned. There were no studies that focused specifically on Queensland, South Australia, Western Australia or Tasmania, and none in New South Wales where the highest population of Aboriginal people live [93]. Further studies are needed with Aboriginal and Torres Strait Islander communities living within these geographic areas.

No studies were found comparing differences of the health impact of racism between Aboriginal people and Torres Strait Islander people. Comparative research could provide more understanding of possible variations in factors such as the most prevalent type of racism by location or differences in the impact of racism on health outcomes in different Aboriginal and Torres Strait Islander communities.

The quality of the included studies was assessed by two authors (CK and UNY) using the Joanna Briggs Institute tool [49, 50] and was found to be moderate to high quality. One of the main strengths of the included articles was their robust sample sizes. Although most had a cross-sectional design, four of them were prospective cohorts, which helped establish temporality. Moreover, from the included papers, three articles [34, 53, 54] measured health outcomes according to the severity of the exposition and their findings suggest a dose-effect association. In line with this, the study by Priest et al., (2012) found a higher and significant association between carer’s and householder’s experience of racism and children presenting 2–4 illnesses during the last two weeks compared to children presenting 0–1 illness [94]. Similarly, a study including 40,000 households in the UK found that progressive exposure to racial discrimination is associated with increased adverse long-term effects on mental wellbeing [95]. The consistency of our results with previous demonstrated dose-response association, adds supporting evidence to infer that racism leads to negative health outcomes [96, 97].

### Strengths and limitations

Strengths of the present study include i) the use of meticulous database search and following standard guidelines during the review process, ii) use of the well-developed protocol with including members from indigenous community (authors KBB and JC), iii) the inclusion of four electronic databases in our search strategy plus manual search on the websites of relevant organisations and snowball technique, iv) a quality appraisal of the included studies using a widely used tool [49, 50].

One limitation of the studies included that nearly all of the studies measured the racism exposure within a 12-month timeframe and used a 4-week timeframe. Although asking explicit timeframes is deemed to be cognitively easier [98], restricting the time period can cause bias [99] since detrimental effects of racism could take a long time to exhibit and participants may not have reported traumatic experiences outside the given timeframe [61]. Another limitation was that although the studies included large sample sizes, the samples were not necessarily representative of Aboriginal and Torres Strait Islander communities. More than 1 article [51, 52, 54, 55] used the same study as a sample source, which limits the generalisability of the results [100]. Despite attempts to perform this review as comprehensively as possible, we might not have identified all studies available on the topic. Only quantitative studies were included, and the search process may have been subject to publication bias. Furthermore, no researchers or experts were contacted for additional studies meeting inclusion criteria that we might have missed.

Contrary to Aboriginal and Torres Strait Islander people’s definition of health, the approach used in this review separated physical from behavioural and mental health outcomes and didn’t evaluate cultural or community wellbeing. This approach was taken since we understand that this method facilitates the statistical analysis and demonstration of the impacts of racism on measurable and objective outcomes. Our research team included two members who are representatives of Aboriginal and Torres Strait Islander communities and with extensive expertise in this research field. However, this limitation highlights the need to include Indigenous methodologies in further studies to ensure that the methods and data analysis better represent Aboriginal and Torres Strait Islander people’s health conception [101–103]. Moreover, and even more importantly, it highlights how Indigenous participation in policy and program development is paramount to ensure that research results are translated into culturally accurate interventions that effectively improve Aboriginal and Torres Strait Islander people’s wellbeing.

## Conclusion

Our study findings indicate that racism is strongly related to adverse effects on the mental health wellbeing. Furthermore, it was found that racism is also negatively associated with general health self-perception whilst its direct association with more specific physical health conditions might be underestimated due to lack of studies. Based on our findings, we suggest the need for the implementation of strategies to address all forms of racism against Aboriginal and Torres Strait Islander peoples as part of the efforts to bridge the health inequity in Australia. Hence, we believe that public health practitioners and policymakers could use our findings as guiding evidence to address racism in partnership with Aboriginal and Torres Strait Islander communities without any delay. We also argue the need for effective implementation of strategies against racism. More research is needed that understands racism and its impacts from an Aboriginal and Torres Strait Islander definition of health and wellbeing. Furthermore, research using longitudinal designs to study the impacts of racism on health outcomes and studies comparing outcomes among states and territories and studies comparing outcomes among states and territories is needed. Aboriginal and Torres Strait Islander-led research studies would also guide self-determined and culturally safe, tailored, and effective interventions for Aboriginal and Torres Strait Islander people.

## Supplementary Information

**Additional file 1.** PRISMA extension for Scoping Reviews (PRISMA-SCR) checklist.

## Data Availability

The datasets generated and analysed during the current study are available upon submitting a reasonable request to the corresponding author.

## Abbreviations

BMI: Body mass index
CCTs: Controlled clinical trials
COPD: Chronic obstructive pulmonary disease
DALYs: Disability – adjusted life years
DRUID: Diabetes and related conditions in indigenous people in the darwin region
LSIC: Longitudinal study of indigenous children
PRISMA: Preferred reporting items for systematic review
PRISMA-SCR: PRISMA extension for scoping reviews
RCTs: Randomized controlled trials

## References

[1] Berman G, Paradies Y (2010). Racism, disadvantage and multiculturalism: towards effective anti-racist praxis. Ethn Racial Stud.

[2] Paradies YC (2006). Defining, conceptualizing and characterizing racism in health research. Crit Public Health.

[3] Larson A, Gillies M, Howard PJ, Coffin J (2007). It’s enough to make you sick: the impact of racism on the health of Aboriginal Australians. Aust N Z J Public Health.

[4] Jones CP. Invited commentary: “Race,” racism, and the practice of epidemiology. Am J Epidemiol. 2001;154(4):299–304. 10.1093/aje/154.4.299 11495851

[5] Krieger N, Rowley DL, Herman AA, Avery B, Phillips MT (1993). Racism, sexism, and social class: implications for studies of health, disease, and well-being. Am J Prev Med.

[6] Harris R, Tobias M, Jeffreys M, Waldegrave K, Karlsen S, Nazroo J (2006). Racism and health: the relationship between experience of racial discrimination and health in New Zealand. Soc Sci Med.

[7] Harrell CJ, Burford TI, Cage BN, Nelson TM, Shearon S, Thompson A, Green S. Multiple pathways linking racism to health outcomes. Du Bois Rev. 2011;8(1):143–57. 10.1017/S1742058X11000178 PMC3328094 22518195

[8] Paradies Y, Ben J, Denson N, Elias A, Priest N, Pieterse A, Gupta A, Kelaher M, Gee G (2015). Racism as a determinant of health: a systematic review and meta-analysis. PLoS One.

[9] Jackson JS, Knight KM, Rafferty JA (2010). Race and unhealthy behaviors: chronic stress, the HPA Axis, and physical and mental health disparities over the life course. Am J Public Health.

[10] Schwelb E (1966). The international convention on the elimination of all forms of racial discrimination. Int Comp Law Q.

[11] United Nations. United Nations Universal Declaration of Human Rights 1948. Off High Com Hum Rights 1948.

[12] UN [United Nations] (2015). The 2007 United Nations declaration on the rights of indigenous peoples. Austrian Rev Int Eur Law Online.

[13] Findling MG, Casey LS, Fryberg SA, Hafner S, Blendon RJ, Benson JM, et al. Discrimination in the United States: experiences of native Americans. Health Serv Res. 2019. 10.1111/1475-6773.13224 PMC6864378 31657013

[14] Environics Institute. Urban Aboriginal peoples study: Winnipeg report. 2011.

[15] Cormack D, Harris R, Stanley J. Māori experiences of multiple forms of discrimination: findings from Te Kupenga 2013. Kotuitui. 2020.

[16] Agüero OA (2002). Sociedades indígenas, racismo y discriminación. Horizontes Antropológicos.

[17] National Health and Medical Research Council. Ethical conduct in research with Aboriginal and Torres Strait Islander peoples and communities: Guidelines for researchers and stakeholders. Canberra; 2018. doi:10.1002/ev.1688, 1994.

[18] AIATSIS. Indigenous Australians: Aboriginal and Torres Strait Islander people. 2018. https://aiatsis.gov.au/explore/articles/indigenous-australians-aboriginal-and-torres-strait-islander-people.

[19] ACT Council of Social Service. Gulanga Good Practice Guides Preferences in terminology when referring to Aboriginal and/or Torres Strait Islander peoples. 2016;:7.

[20] Walter M, Academy of the Social Sciences in Australia., Australian Bureau of Statistics. Lives of diversity: indigenous Australia. 2008. http://www.assa.edu.au/publications/occasional_papers/2008_CS2.php.

[21] Paradies Y. Colonisation, racism and indigenous health. J Popul Res. 2016;33(1):83–96.

[22] Australian Bureau of Statistics. National Aboriginal and Torres Strait Islander Social Survey. Natl Aborig Torres Strait Islander Soc Surv. 2015.

[23] Australia R. 2018 Australian Reconciliation Barometer 2018.

[24] Shirodkar S. Bias against Indigenous Australians: Implicit Association Test results for Australia 2018;22:3–34.

[25] Australian Institute of Health and Welfare. Deaths in Australia. Canberra; 2019. doi: PHE 229.

[26] Australian Institute of Health and Welfare, Al-Yaman F. The Australian Burden of Disease Study: impact and causes of illness and death in Aboriginal and Torres Strait Islander people, 2011 Syudy series no. 6 Cat no. BOD 7. Public Heal Res Pract. 2017. 10.17061/phrp2741732 29114712

[27] Australian Health Ministers’ Advisory Council. Aboriginal and Torres Strait Islander Health Performance Framework. 2014. https://www.pmc.gov.au/sites/default/files/publications/Aboriginal_and_Torres_Strait_Islander_HPF_2014 - edited 16 June2015.pdf.

[28] Australian Human Rights C. Close the Gap: Indigenous Health Equality Summit Statement of Intent. Canberra Aust Hum Rights Comm. 2008.

[29] Commonwealth of Australia. Closing the Gap Prime Minister’s Report 2017. Commonw Aust Dep Prime Minist Cabinet. 2017;:112. doi:10.5117/9789053565742.

[30] Davis M. New agreement won’t deliver the change indigenous Australians need. The Sydney Morning Herald 2020.

[31] Bond C. The “new” closing the gap is about buzzwords, not genuine change for indigenous Australian. The conversation 2020.

[32] Came H, Griffith D (2018). Tackling racism as a “wicked” public health problem: enabling allies in anti-racism praxis. Soc Sci Med.

[33] Brown AF, Ma GX, Miranda J, Eng E, Castille D, Brockie T, Jones P, Airhihenbuwa CO, Farhat T, Zhu L, Trinh-Shevrin C (2019). Structural interventions to reduce and eliminate health disparities. Am J Public Health.

[34] Markwick A, Ansari Z, Clinch D, McNeil J (2019). Perceived racism may partially explain the gap in health between Aboriginal and non-Aboriginal Victorians: a cross-sectional population based study. SSM - Popul Heal.

[35] UN. Transforming Our World: the 2030 Agenda for Sustainable Development United Nations United Nations Transforming Our World: the 2030 Agenda for Sustainable Development. A/RES/70/1. 2015.

[36] Australian Medical Association. AMA 2018 report card on Indigenous health. 2018.

[37] Williams DR, Lawrence JA, Davis BA. Racism and health: evidence and needed research. Annu Rev Public Health. 2019;40:105–25. 10.1146/annurev-publhealth-040218-043750 PMC6532402 30601726

[38] Stanley LR, Swaim RC, Kaholokula JK, Kelly KJ, Belcourt A, Allen J. The imperative for research to promote health equity in indigenous communities. Prev Sci. 2020;21(1):13–21. 10.1007/s11121-017-0850-9 PMC5936666 29110278

[39] Kairuz CA, Casanelia LM, Bennett-Brook K, Coombes J, Narayan YU (2020). Impact of racism and discrimination on the physical and mental health among Aboriginal and Torres Strait islander peoples living in Australia: a protocol for a scoping review. Syst Rev.

[40] Moher D, Liberati A, Tetzlaff J, Altman DG, Altman D, Antes G (2009). Preferred reporting items for systematic reviews and meta-analyses: the PRISMA statement. PLoS Med.

[41] Arksey H, O’Malley L (2005). Scoping studies: towards a methodological framework. Int J Soc Res Methodol Theory Pract.

[42] Levac D, Colquhoun H, O’Brien KK (2010). Scoping studies: advancing the methodology. Implement Sci.

[43] Marshall IJ, Marshall R, Wallace BC, Brassey J, Thomas J (2019). Rapid reviews may produce different results to systematic reviews: a meta-epidemiological study. J Clin Epidemiol.

[44] Hupe M (2019). EndNote X9. J Electron Resour Med Libr.

[45] Bogdanos DP, Smyk DS, Rigopoulou EI, Mytilinaiou MG, Heneghan MA, Selmi C, Eric Gershwin M (2012). Twin studies in autoimmune disease: genetics, gender and environment. J Autoimmun.

[46] Cooper GS, Miller FW, Pandey JP (1999). The role of genetic factors in autoimmune disease: implications for environmental research. Environ Health Perspect.

[47] Goris A, Liston A. The immunogenetic architecture of autoimmune disease. Cold Spring Harb Perspect Biol. 2012;4(3). 10.1101/cshperspect.a007260. 10.1101/cshperspect.a007260 PMC3282406 22383754

[48] Peters MDJ, Godfrey CM, McInerney P, Munn Z, Tricco AC, Khalil H. Chapter 11: scoping reviews (2020 version). In: Joanna Briggs Institute Reviewer’s Manual. 2020.

[49] Moola S, Munn Z, Tufanaru C, Aromataris E, Sears K, Sfetcu R, Currie M, Lisy K, Qureshi R, Mattis P, Mu P. Chapter 7: Systematic reviews of etiologyand risk. In: Aromataris E, Munn Z (Editors). JBI Manual for Evidence Synthesis. JBI, 2020. Available from 10.46658/JBIMES-20-08.

[50] Moola S, Munn Z, Tufanaru C, Aromataris E, Sears K, Sfetcu R, et al. Checklist for analytical cross sectional studies. Joanna Briggs Inst Rev Man. 2017;6. 10.17221/96/2009-CJGPB.

[51] Cave L, Cooper MN, Zubrick SR, Shepherd CCJ (2019). Caregiver-perceived racial discrimination is associated with diverse mental health outcomes in Aboriginal and Torres Strait islander children aged 7-12 years. Int J Equity Health.

[52] Macedo DM, Smithers LG, Roberts RM, Paradies Y, Jamieson LM. Effects of racism on the socio-emotional wellbeing of Aboriginal Australian children. Int J Equity Health. 2019;18(1):1–0. 10.1186/s12939-019-1036-9 PMC6706881 31438974

[53] Temple JB, Kelaher M, Paradies Y. Experiences of racism among older Aboriginal and Torres Strait Islander People: prevalence, sources, and association with mental health. Can J Aging. 2020; 39(2):178–89. 10.1017/S071498081900031X. 10.1017/S071498081900031X 31230607

[54] Shepherd CCJ, Li J, Cooper MN, Hopkins KD, Farrant BM (2017). The impact of racial discrimination on the health of Australian indigenous children aged 5-10 years: analysis of national longitudinal data. Int J Equity Health.

[55] Cave L, Shepherd CCJ, Cooper MN, Zubrick SR. Racial discrimination and the health and wellbeing of Aboriginal and Torres Strait islander children: does the timing of first exposure matter? SSM - Popul heal. 2019. 10.1016/j.ssmph.2019.100492 PMC6804701 31649999

[56] Paradies YC, Cunningham J (2012). The DRUID study: exploring mediating pathways between racism and depressive symptoms among indigenous Australians. Soc Psychiatry Psychiatr Epidemiol.

[57] Paradies YC, Cunningham J. The DRUID study: racism and self-assessed health status in an indigenous population. BMC Public Health. 2012;12(1). 10.1186/1471-2458-12-131. 10.1186/1471-2458-12-131 PMC3305656 22333047

[58] Priest NC, Paradies YC, Gunthorpe W, Cairney SJ, Sayers SM (2011). Racism as a determinant of social and emotional wellbeing for Aboriginal Australian youth. Med J Aust.

[59] Kelaher MA, Ferdinand AS, Paradies Y (2014). Experiencing racism in health care: the mental health impacts for Victorian Aboriginal communities. Med J Aust.

[60] Priest N, Paradies Y, Stewart P, Luke J. Racism and health among urban Aboriginal young people. BMC Public Health. 2011;11 June 2014. 10.1186/1471-2458-11-568 PMC3146875 21756369

[61] Paradies YC, Cunningham J. Development and validation of the measure of indigenous racism experiences (MIRE). Int J Equity Health. 2008;7(1). 10.1186/1475-9276-7-9. 10.1186/1475-9276-7-9 PMC2359753 18426602

[62] Hannes K, Lockwood C, Pearson A. A comparative analysis of three online appraisal instruments’ ability to assess validity in qualitative research. Qual Health Res. 2010;20(12):1736–43. 10.1177/1049732310378656 20671302

[63] Munn Z, Moola S, Riitano D, Lisy K (2014). The development of a critical appraisal tool for use in systematic reviews addressing questions of prevalence. Int J Heal Policy Manag.

[64] Ramaswamy M, Kelly PJ. Institutional racism as a critical social determinant of health [Editorial]. Public Health Nurs. 2015;32(4):285–6. 10.1111/phn.12212. 10.1111/phn.12212 26199054

[65] Cobbinah SS, Lewis J. Racism & Health: a public health perspective on racial discrimination. J Eval Clin Pract. 2018;24(5):995–8. 10.1111/jep.12894 29508479

[66] Hertz RP, Unger AN, Cornell JA, Saunders E (2005). Racial disparities in hypertension prevalence, awareness, and management. Arch Intern Med.

[67] Maura J, de Mamani A (2017). Mental health disparities, treatment engagement, and attrition among racial/ethnic minorities with severe mental illness: a review. J Clin Psychol Med Settings.

[68] Houston S, Legge D. Aboriginal health research and the national aboriginal health strategy. Aust J Public Health. 1992;16(2):114–5. 10.1111/j.1753-6405.1992.tb00037.x 1391150

[69] Commonwealth of Australia. National Aboriginal and Torres Strait Islander Health Plan 2013–2023. 2013. http://www.health.gov.au/internet/main/publishing.nsf/content/B92E980680486C3BCA257BF0001BAF01/$File/health-plan.pdf.

[70] Australian Indigenous HealthInfoNet. Social and Emotional Wellbeing. 2020.

[71] Kirkinis K, Pieterse AL, Martin C, Agiliga A, Brownell A. Racism, racial discrimination, and trauma: a systematic review of the social science literature. Ethn Health 2018;0:1–21. doi:10.1080/13557858.2018.1514453, 26, 3. 10.1080/13557858.2018.1514453 30165756

[72] Britt-Spells AM, Slebodnik M, Sands LP, Rollock D (2018). Effects of perceived discrimination on depressive symptoms among black men residing in the United States: a meta-analysis. Am J Mens Health.

[73] Assari S, Lankarani MM, Caldwell CH. Discrimination increases suicidal ideation in black adolescents regardless of ethnicity and gender. Behav Sci. 2017;7(4):75. 10.3390/bs7040075 PMC5746684 29113117

[74] Priest N, Truong M, Chong S, Paradies Y, King TL, Kavanagh A, Olds T, Craig JM, Burgner D. Experiences of racial discrimination and cardiometabolic risk among Australian children. Brain Behav Immun. 2020;87:660–5. 10.1016/j.bbi.2020.02.012 32119900

[75] Cuffee YL, Hargraves JL, Allison J. Exploring the association between reported discrimination and hypertension among African Americans: a systematic review. Ethn Dis. 2012;22(4):422–32. 23140072

[76] Dolezsar CM, McGrath JJ, Herzig AJM, Miller SB (2014). Perceived racial discrimination and hypertension: a comprehensive systematic review. Health Psychol.

[77] Markwick A, Ansari Z, Clinch D, McNeil J (2019). Experiences of racism among Aboriginal and Torres Strait islander adults living in the Australian state of Victoria: a cross-sectional population-based study. BMC Public Health.

[78] Read UM, Karamanos A, Silva MJ, Molaodi OR, Enayat ZE, Cassidy A (2018). The influence of racism on cigarette smoking: longitudinal study of young people in a British multiethnic cohort. PLoS One.

[79] Desalu JM, Goodhines PA, Park A (2019). Racial discrimination and alcohol use and negative drinking consequences among black Americans: a meta-analytical review. Addiction..

[80] Gibbons FX, Gerrard M, Cleveland MJ, Wills TA, Brody G (2004). Perceived discrimination and substance use in African American parents and their children: a panel study. J Pers Soc Psychol.

[81] Winter T, Riordan BC, Surace A, Scarf D. Association between experience of racial discrimination and hazardous alcohol use among Māori in Aotearoa New Zealand. Addiction. 2019;114(12):2241–6. 10.1111/add.14772 31386231

[82] Gerrard M, Stock ML, Roberts ME, Gibbons FX, O’Hara RE, Weng CY (2012). Coping with racial discrimination: the role of substance use. Psychol Addict Behav.

[83] Winstanley M, van der Sterren A, Knoche D (2012). 8.2 history of tobacco use among Aboriginal peoples and Torres Strait islanders. In: tobacco in Australia: Facts & Issues.

[84] Passey ME, Gale JT, Sanson-Fisher RW (2011). “It’s almost expected”: rural Australian Aboriginal women’s reflections on smoking initiation and maintenance: a qualitative study. BMC Womens Health.

[85] Durey A, Thompson SC, Wood M (2012). Time to bring down the twin towers in poor Aboriginal hospital care: addressing institutional racism and misunderstandings in communication. Intern Med J.

[86] Henry BR, Houston S, Mooney GH (2004). Institutional racism in Australian healthcare: a plea for decency. Med J Aust.

[87] Paine SJ, Stanley J. Caregiver experiences of racism are associated with adverse health outcomes for their children: a cross-sectional analysis of data from the New Zealand Health Survey. Crit Public Health. 2020;30(5):509–20.

[88] Kelly Y, Becares L, Nazroo J (2013). Associations between maternal experiences of racism and early child health and development: findings from the UK millennium cohort study. J Epidemiol Community Health.

[89] Heard-Garris NJ, Cale M, Camaj L, Hamati MC, Dominguez TP. Transmitting Trauma: a systematic review of vicarious racism and child health. Soc Sci Med. 2018;199:230–40. 10.1016/j.socscimed.2017.04.018 28456418

[90] Currie CL, Copeland JL, Metz GA, Chief Moon-Riley K, Davies CM (2020). Past-year racial discrimination and allostatic load among indigenous adults in Canada: the role of cultural continuity. Psychosom Med.

[91] Houkamau CA, Stronge S, Sibley CG. The prevalence and impact of racism toward indigenous Maori in New Zealand. Int Perspect Psychol Res Pract Consult. 2017;6(2):61–80.

[92] Beyond Blue. Discrimination against indigenous Australians. 2014. https://www.beyondblue.org.au/docs/default-source/research-project-files/bl1337-report%2D%2D-tns-discrimination-against-indigenous-australians.pdf?sfvrsn=2.

[93] Australian Bureau of Statistics. Estimates of Aboriginal and Torres Strait Islander Australians, June 2016. 2018. https://www.abs.gov.au/ausstats/abs@.nsf/mf/3238.0.55.001. Accessed 24 Jul 2020.

[94] Priest N, Paradies Y, Stevens M, Bailie R (2012). Exploring relationships between racism, housing and child illness in remote indigenous communities. J Epidemiol Community Health.

[95] Wallace S, Nazroo J, Bécares L (2016). Cumulative effect of racial discrimination on the mental health of ethnic minorities in the United Kingdom. Am J Public Health.

[96] Fedak KM, Bernal A, Capshaw ZA, Gross S (2015). Applying the Bradford Hill criteria in the 21st century: how data integration has changed causal inference in molecular epidemiology. Emerg Themes Epidemiol.

[97] Lucas RM, McMichael AJ. Association or causation: evaluating links between “environment and disease.”. Bull World Health Organ. 2005;83:792–5. PMC2626424 16283057

[98] Blank R, Dabady M, Citro C. Measuring Racial Discrimination. 2004.

[99] Utsey SO, Ponterotto JG (1996). Development and validation of the index of race-related stress (IRRS). J Couns Psychol.

[100] Kukull WA, Ganguli M. Generalizability: The trees, the forest, and the low-hanging fruit. Neurology. 2012;78(23):1886–91. 10.1212/WNL.0b013e318258f812 PMC3369519 22665145

[101] Ryder C, Mackean T, Coombs J, Williams H, Hunter K, Holland AJA, Ivers RQ (2020). Indigenous research methodology – weaving a research interface. Int J Soc Res Methodol.

[102] Durie M. Exploring the Interface between science and indigenous knowledge. In 5th APEC Research and Development Leaders Forum, Christchurch, New Zealand. 2004. (pp. 2–21).

[103] Bartlett C, Marshall M, Marshall A (2012). Two-eyed seeing and other lessons learned within a co-learning journey of bringing together indigenous and mainstream knowledges and ways of knowing. J Environ Stud Sci.

